# Staying home saves lives, really!

**DOI:** 10.1007/s12076-022-00316-6

**Published:** 2022-08-31

**Authors:** Maurizio Malpede, Soheil Shayegh

**Affiliations:** 1 Bocconi University and University of Verona, Milan, Italy; 2 RFF-CMCC European Institute on Economics and the Environment (EIEE), Centro Euro-Mediterraneo sui Cambiamenti Climatici, Lecce, Italy

**Keywords:** Coronavirus, COVID-19, Crime, Quarantine, I18, J12, K14

## Abstract

When coronavirus disease (COVID-19) was spreading worldwide, many national and local governments started to impose socially restrictive measures to limit the spread of the virus. Such quarantine measures in different cities worldwide have brought a new trend in public safety improvement and crime reduction. Using daily crime reports in the U.S., this paper evaluates the immediate unintended effects of shelter-in-place orders on different crime categories using fine-grained spatial units (i.e., neighborhoods) rather than entire cities, states, or countries. Results for San Francisco suggest an immediate drop of between 10 and 20% points in the total number of crimes after one month from the introduction of the restrictions. In particular, we show that while theft, homicide, and traffic accidents have fallen sharply, domestic violence incidents and weapon possession offences were not affected by the lockdown. The results are robust to the inclusion of spatial and temporal dependence.

## Introduction

Coronavirus disease (COVID-19) spread fast across the globe after its initial outbreak in Wuhan, China, in December 2019. On January 30 of the following year, the World Health Organization (WHO) declared a public health concern. On March 11, the WHO finally recognized the COVID-19 outbreak as a global pandemic urging all countries to step up their efforts to prevent the spread of the virus.

During the early phases of the diffusion, while the search for medical treatment and prevention (e.g., vaccine) was still going on, many local and national governments introduced non-pharmaceutical interventions (NPIs) in the form of strict social restrictions to curb the spread of the virus in their communities with varying degrees of success. As a result, millions of people worldwide experienced different degrees of travel restrictions and self-isolation in their homes. The effectiveness of such policies in reducing death and hospitalization have been the subject of long academic an policy debates but recent studies seem to suggest that in countries with higher degree of subnational autonomy (e.g., the U.S.), the negative consequences of the pandemic have been more severe (Chiara 2021).

Nevertheless, the first official lockdown was introduced in the city of Wuhan on January 23, 2020, followed by travel restrictions in other cities within Hubei province. Large countries like India, South Africa, and many European countries have introduced a country-wide lockdown outside China.

For what concerns the US experience, during the initial diffusion of the virus, the federal government did not implement a nationwide quarantine. This has led many states to impose state-wide restrictions. On a more local level, while some major cities like New York had an initially slow response to the fast spread of the virus, other communities like those in California did not hesitate to take some of the strictest public health orders. On March 16, six communities in the San Francisco Bay Area issued a shelter-in-place order that immediately affected the social lives of millions of people living in that area. The order was followed after a few days by a similar but state-wide order from the governor of California.

The broader socioeconomic impacts of the current pandemic and the subsequent restrictive measures are yet to be investigated (Wilder-Smith and Freedman 2020). However, there are clear indications from early observations that social distancing and widespread closures, along with restrictive measures such as national and local quarantine orders, have been effective in slowing the spread of the virus (Lau et al. 2020). Furthermore, enforcing quarantine measures in different cities across the world has brought a new trend in public safety improvement, and crime reduction (Jacobs and Barrett 2020; www.kansascitymag.com; www.latimes.com ). That being said, there is a well-documented risk that as more people are forced to work remotely, criminal organizations seize this opportunity and turn to cyberspace for a wide range of criminal activities, from fraud and forgery in the procurement of much-needed medical equipment to organized property crime by impersonation of representatives of public authorities (EUROPOL 2020).

Recent literature has quantified the immediate decline in criminal incidents following the quarantine in the US (Abrams 2021; Boman and Gallupe 2020). Specifically, it has been shown that shelter-in-place restrictions were associated with a reduction of at least 35% in overall crime, which was especially pronounced for most violent crimes (Abrams 2021). However, there was no decline in homicides and shootings and an increase in non-residential burglary and car theft in most cities of the US. On the other hand, the drop in the number of crimes was due to the decline in minor offenses (Boman and Gallupe 2020).

In a related paper, considering 27 cities across 23 countries worldwide, it was shown that crime decreased by 37% after governments issued stay-at-home orders (Boman and Mowen 2021). The decline rates varied substantially across crime types, with some crimes decreasing more than others. Specifically, property-based crimes decreased substantially, but homicide was relatively unchanged. Focusing on domestic violence in the city of Chicago and using cell phone block-level activity data, the staying-at-home order increased time spent at home, leading to a subsequent increase in domestic violence-related calls for police service (Bullinger et al. 2020). Similarly, focusing on Mexico city, sexual offenses and domestic violence were shown to decline during the COVID-19 lockdown. At the same time, femicide rates remained constant during the pandemic (Hoehn-Velasco et al. 2021).^1^

Concerning the long-term effects of COVID-19 restrictions on the mobility of people, as stay-at-home orders eased, crime increased to pre-COVID-19 historical trends (Boman and Mowen 2021). The results show that sexual offenses, lapses in alimony, and domestic violence decline during the COVID-19 lockdown and then return to pre-pandemic levels by the fall.

This paper focuses on the effects of restrictive measures on the number of crimes in general and on different categories of criminal activities in specific by exploiting daily crime level data at neighborhood levels for two major U.S. cities. In particular, we study the effects of the shelter-in-place order on criminal activity using fine-grained spatial units (i.e., neighborhoods). We contributes to the existing literature on the impacts of COVID-19 related restrictions on crimes which have considered entire cities, states, or countries.

We provide a robust quantitative assessment to address the following questions: have shelter-in-place (henceforth SIP) policies caused a reduction in the number of incidents? And if they did, what types of criminal activities were mostly affected by these restrictive policies? Finally, we ask how the reported domestic violence cases have been impacted by shelter-in-place?

To assess the impact of the SIP policy first introduced in the cities of San Francisco and Oakland, we adopt an event study approach using official historical geo-localized daily data on the criminal activity before and after the introduction to people’s mobility.

To make the empirical procedure robust, we first consider the number of daily reported COVID-19 infections in the city of San Francisco along with weather data which include the average daily temperature, precipitation, and heating degree days. In addition, we also include temporal lags of the weather controls since there exists a correlation between city crimes and weather variations (Cohn 1990; Blakeslee and Fishman 2018). Moreover, to account for time-varying factors which may independently affect criminal activity, the analysis controls for the day of the week and the month of the year. In addition, we include neighborhood by time fixed effects in order to account for any time varying difference across neighborhoods within the city.

Finally, since crime is an excellent example of a spatially correlated process (Anselin et al. 2000; Elhorst 2014), we also adopt a simple method to account for spatial autocorrelation in the data following the recent literature on spatial determinants of conflicts (Harari and La Ferrara 2018).

**Fig. 1:**
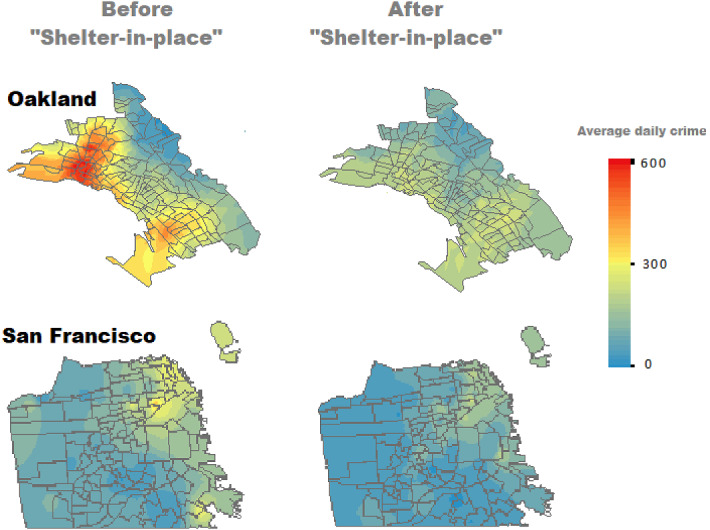
Daily the number of crimes reduced sharply in both Oakland and San Francisco within a month following the shelter-in-place order of March 16th, 2020

**Fig. 2:**
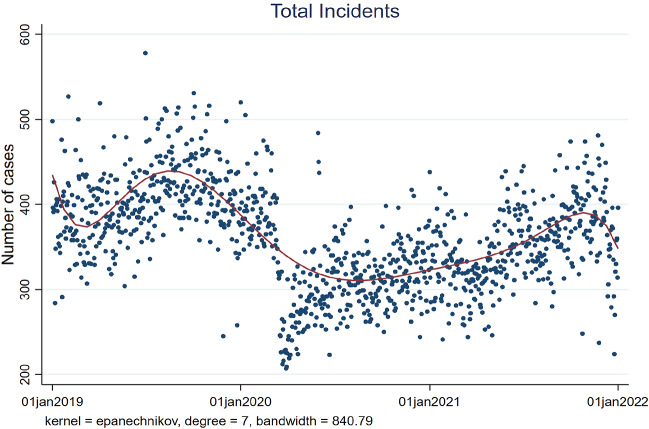
Total reports of criminal incidents from 01-01-2019 to 01-01-2022 in San Francisco. As the restrictions eased, the number of incidents slightly approached its pre-covid trend

We first show that the total number of crimes in the two large cities in the US was negatively affected by the intervention to stop the diffusion of the virus. In particular, the daily number of total criminal incidents declined by more than 30% after one month from the introduction of the SIP in San Francisco. Traffic crimes, thefts, homicides, and vandalism were mostly affected by the restriction. On the other hand, we resent no immediate reduction in domestic violence after two weeks from the restrictive order. However, we find the reduction in criminal activity to be significant in the short term only. Indeed, as the restrictions eased, the crimes slowly approached their historical trends. However, as shown in Figs. 2 and 4, it appears that crimes in San Francisco even after the SIP order and the consequent return to normal life were slightly below their pre-covid levels.

**Fig. 3:**
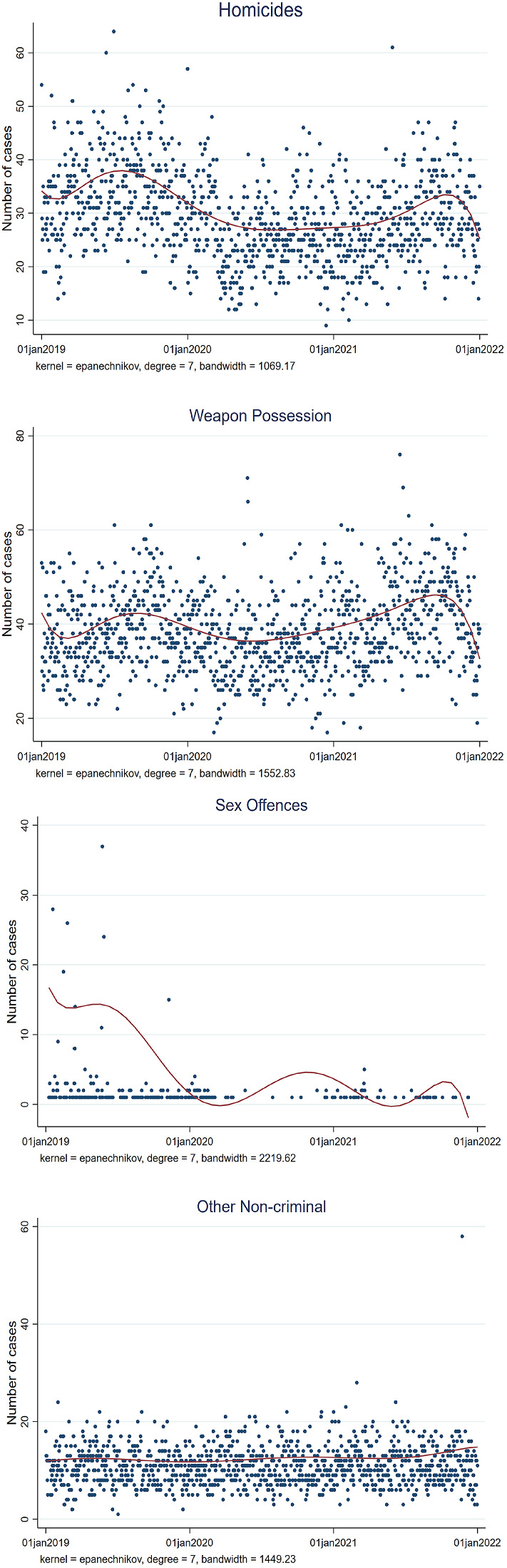
Reports of criminal incidents from 01-01-2020 to 01-01-2022 in San Francisco selected by type of crime

**Fig. 4:**
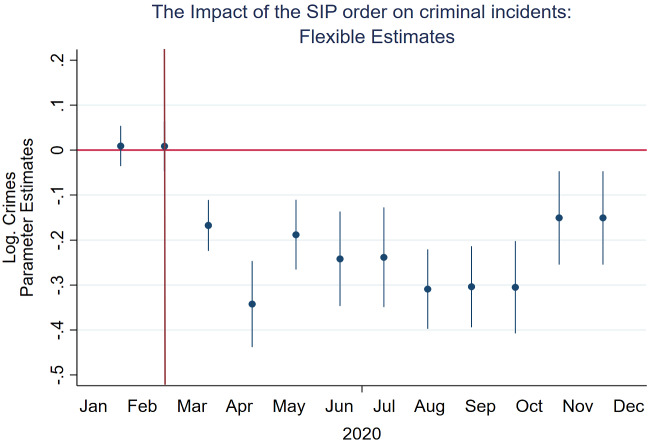
Impact of the Shelter-in-Place order on the natural logarithm of criminal incidents per month from 01-01-2019 to 31-12-2020 in San Francisco. This figure presents results of the estimation of Eq. 2. The periods span between January and December 2020, and are observed every other month. In this specification, we evaluate the effects of the SIP order for each month. Coefficients are reported with confidence intervals based on standard errors, clustered at the neighborhood level

In addition to a standard fixed effect model we also estimate a model that includes spatial and temporal autoregressive terms. We show that the decline of all criminal activities falls between 10 and 20% points. While corroborating the baseline results, these results also highlight the spatial correlation of criminal activities.

The remainder of the paper is organized as follows. Section 2 describes the data used to estimate the impact of the forced lockdown on the number of crimes. Section 3 describes the empirical strategy. Section 4 shows the baseline results from the analysis and results for different types of crime. Finally, Sect. 5 concludes.

## Data

Official historical data on daily incident reports for the city of San Francisco is provided by the San Francisco Police Department. Incidents are updated daily and are divided into 33 different categories^2^ and are recorded for each neighborhood of San Francisco. Figure 2 shows the daily total number of crimes in San Francisco and a local polynomial smooth fit on the data. As it is established, the trend started a steep decline after introducing the shelter-in-place order on March 16.^3^

Along with crime data for San Francisco, we also use weather data. In particular, we use the average daily temperature and level of precipitations since May 2018 registered in San Francisco from the National Weather Service (National Weather Service 2020).

Historical data for the city of Oakland provided by the Oakland Police department covers only the last 90 days and, therefore, can not be used to construct the historical assessment. We use only these data for demonstration and comparison to our main results from San Francisco.

### Definition of pre and post shelter-in-place order in San Francisco

To allow for a meaningful comparison of the criminal cases before and after the onset of the shelter-in-place order, we consider the historical daily registered number of incidents for the city of San Francisco from May 1, 2018, until March 16, 2020, as the pre-policy data and the records after March 16, 2020, until March 28, 2020, as post-policy data. Incident reports are also geo-localized. This allows us to compare the number of crimes for each neighborhood to its historical average in the same period.

## Econometric estimation

We use the following estimation setup for evaluating the impact of policy intervention on the daily number of crimes:

1 $$\begin{aligned} y_{i,t} = \alpha + \beta \cdot I^{post} + \phi _{1}{\mathbf {X}}_t + \phi _{2}{\mathbf {X}}_{t-1} + \delta _t +\gamma _{it} +\omega _d +\epsilon _{i,t} \end{aligned}$$

where, dependent variable $y_{i,t}$ stands for the natural logarithm of the reported number of incidents on day *t*, in the neighborhood *i*. The natural logarithm allows for estimating the effects of the restrictions on the percentage of criminal activities in each neighborhood. $I^{post}$ is an indicator variable that takes a value of 0 before the implementation of the shelter-in-place policy against the diffusion of the COVID-19 that occurred on March 16, 2020, and a value of 1 for days after March 16. The coefficient of interest $\beta$ measures the impact of the shelter-in-place order on criminal activity in neighbor *i*. The term ${\mathbf {X}}_t$ represents vectors of daily weather conditions, while the term ${\mathbf {X}}_{t-1}$ represents the time lag of the weather controls.

Weather variables include the average daily temperature and precipitations recorded by the National Weather Service. The inclusion of weather controls is supported by several studies showing a positive correlation between temperature and crime rate, particularly violent types of crime (Tiihonen et al. 2017; Ranson 2014). Variable $\delta _t$ indicates a full set of day-of-the-year fixed effects while $\gamma _{it}$ represents the neighborhood by time fixed effects. These control variables are crucial to estimating the causal impact of restrictive policy measures on daily crime activity because they capture day-specific regularities in each neighborhood (e.g., Christmas holidays and thanksgiving). In addition, they allow us to capture any differences in neighborhoods over time.

Finally, $\omega _d$ indicates the day of the week fixed effects (this is done to take into account variations in the number of crimes that might occur on a particular day of the week, e.g., on Saturdays, crimes are typically higher than Sundays). The term $\epsilon _{i,t}$ is an iid error term.

### Flexible estimates

Here, we estimate a fully flexible estimating equation that takes the following form:

2 $$\begin{aligned} y_{i,t} = \alpha + \sum _{m=1}^{12} \beta _j \cdot I_t^{m} + \phi _{1}{\mathbf {X}}_t + \phi _{2}{\mathbf {X}}_{t-1} + \delta _t +\gamma _{it} +\omega _d +\epsilon _{i,t} \end{aligned}$$

The only difference from Eq. 1 is that in Eq. 2, rather than considering a post-SIP order indicator variable, we evaluate the effects of the SIP order for each month from January to December 2020. The estimated vectors of $\beta _m$ now reveal the effects of the SIP order on crimes in each month of the year 2020. If no other event occurred in the same period, and the SIP order had a negative effect on criminal activity, then we would expect the estimated $\beta _m$s not to be statistically significant for the months *before* March (i.e., January, and February) while becoming negative and significant starting from March only. Figure 4 shows the estimated $\beta _m$s compared to February 2020 (i.e., the last month before the shelter-in-place order was implemented in San Francisco) along with the associated confidence intervals which confirm our prediction.

### Inclusion of spatial and temporal dependence

To account for spatial correlation in the covariates, we include spatial and temporal lags of the variable of interest, namely the natural logarithm of the number of crimes for each neighborhood on day t. The spatial dependence structure is defined by a symmetric weighting matrix W, and the spatial lag of a variable is obtained by multiplying the matrix W by the vector of observations.

3 $$\begin{aligned} \begin{aligned} y_{i,t}&= \alpha + \beta _1 y_{i,t-1} + \beta _2 {\mathbf {W}}\cdot y_{i,t} + \gamma \cdot I^{post} + \phi _{1,t}{\mathbf {W}} \cdot {\mathbf {X}}_t + \\&+ \phi _{2,t}{\mathbf {W}} \cdot {\mathbf {X}}_{t-1} + \delta _t +\gamma _{it} +\omega _d +\epsilon _{i,t} \end{aligned} \end{aligned}$$

where, as in Eq. 1, $y_{i,t}$ denotes the natural logarithm of reported number of incidents on day *t*, in neighborhood *i*. $I^{post}$ is an indicator variable that takes a value of 0 before the implementation of the shelter-in-place order against the diffusion of the COVID-19 that occurred on March 16, 2020, and a value of 1 for days after March 16.

We estimate this relationship to adjust standard errors for both spatial and serial correlation. The spatial matrix W included in the dynamic model described in Eq. 2 exploits variations in criminal activity occurring in neighborhoods whose centroids are located within 1 kilometer) as "first-degree neighbors." In comparison, grids whose centroids are situated between 1 and 2 kilometers are defined as "second-degree neighbors." Thus, our implicit identifying assumption is that criminal activity occurring in neighborhoods beyond 2 kilometers from the neighborhood i do not affect the number of crimes in the own cell other than by affecting crimes that spill over in space.

Variable $\delta _t$ indicates a full set of day-of-the-year fixed effects while $\gamma _{it}$ represents the neighborhood-specific by time fixed effects. $\omega _d$ indicates the day of the week fixed effects. Finally, the term $\epsilon _{i,t}$ is an iid error term.

The coefficient of interest $\beta$ measures the impact of the shelter-in-place order on criminal activity in the neighborhood *i*. As before, the term ${\mathbf {X}}_t$ represents vectors of daily weather conditions, while the term ${\mathbf {X}}_{t-1}$ represents the time lag of the weather controls.

## Results

The results of our analysis show a substantial reduction in the number of crimes in both San Francisco and Oakland. As mentioned in Sect. 2, the daily historical data are only available for San Francisco; therefore, we report the results of our analysis for San Francisco here. We use Oakland’s 90-day data to construct a visualization comparison with historical trends in San Francisco.

**Table 1:** The impact of the shelter-in-place order on the number of criminal incidents

	Dep. variable: in(total crimes)			
	1	2	3	4
$$I^{\mathrm{post}}$$	– 0.401$$***$$	– 0.361$$***$$	– 0.317$$***$$	– 0.245$$***$$
	(0.034)	(0.039)	(0.034)	(0.033)
Day of week FE	No	Yes	Yes	Yes
Time FE	No	Yes	Yes	Yes
Covid-19 cases	No	No	Yes	Yes
Neighborhood x year FE	No	No	Yes	Yes
Weather controls	No	No	No	Yes
Observations	275,183	275,183	275,183	273,183

This table presents results of the estimation of Eq. 1

The effects of social distancing measures on the natural logarithm of total crimes after one month from the introduction of the SIP in San Francisco. Significant at $$***$$*p* < 0.01,$$**$$*p* < 0.05, $$*$$*p* < 0.1

### Impact of the shelter-in-place order on total crime rate

Figure 1 shows that the number of crimes has plunged abruptly after implementing the order in both cities. Table 1 reports estimates of the impact of the shelter-in-place on the total crime rate as in Eq. 1. The first column does not include any control. Results in this column indicate a reduction of more than 40% in the daily number of crimes after the lockdown. The second column includes controls only for the day of the week (e.g., typically, Police Departments register a higher number of incidents during the weekend). Results in this column indicate a reduction of more than 36% in daily the number of crimes after the lockdown.

Column (3) shows estimates when adding controls from the daily number of COVID-19 cases for the city of San Francisco and neighborhood by time fixed effects^4^. Results in this column indicate a reduction of more than 31% in daily the number of crimes after the lockdown. The coefficients indicating the impact of the shelter-in-place order on total daily reported crimes do not significantly change. Therefore, concerns about the possible selection of unobservables are alleviated.

Finally, column (4) shows estimates when adding weather controls, i.e., average daily temperature, heating degree days, and total precipitations) and their temporal lag. This specification shows our baseline results. Here, we find that the SIP policy reduced total daily crime activity by 24.5% with respect to its historical trend.

We find the reduction in criminal activity to be significant only within a month from the SIP order. Indeed, estimating Eq. (1) for each month specifically, we find that as the restrictions eased, the crimes slowly approached their historical trends (Fig. 4). However, even after the end of the SIP order and the consequent return to normal life, they were slightly below their pre-covid levels.

**Table 2:** The impact of the shelter-in-place order on the number of criminal incidents

	Dep. variable: in (total crimes)			
	1	2	3	4
$$I^{post}$$	– 0.197$$***$$	– 0.173$$***$$	– 0.173$$***$$	– 0.132$$***$$
	(0.021)	(0.022)	(0.022)	(0.021)
$$W \cdot Crimes$$	0.030$$***$$	0.029$$***$$	0.029$$***$$	0.029$$***$$
	(0.004)	(0.004)	(0.004)	(0.004)
Day of week FE	No	Yes	Yes	Yes
Time FE	No	Yes	Yes	Yes
Covid-19 cases	No	No	Yes	Yes
Neighborhood $\times$ year FE	No	No	Yes	Yes
Weather Controls	No	No	No	Yes
Observations	275,183	275,183	275,183	273,183

Inclusion of spatial and temporal dependence

This table presents results of the effects of social distancing measures on the natural logarithm of total crimes after one month from the introduction of the SIP in San Francisco. The model includes spatial and temporal dependence. Significant at $$***$$*p* < 0.01, $$**$$*p* < 0.05, $$*$$*p* < 0.1

### Inclusion of spatial and temporal dependence

Table 2 reports estimates of the impact of the shelter-in-place on the total crime rate as in Eq. 3. The first column does not include any control. Results in this column indicate a reduction of about 20% in the daily number of crimes after the lockdown. These results indicate that, on the one hand, the reduction in criminal activities following the restrictions on people’s mobility is confirmed in a spatial regression model. On the other hand, they point out the possible overestimation of the SIP policy on crime reduction if we estimate a model that does not consider the spatial and temporal dependence of criminal activity within a city.

As for Eq. 1, the second column includes controls only for the day of the week (e.g., typically, Police Departments register a higher number of incidents during the weekend). Results in this column indicate a reduction of more than 17% in the daily number of crimes after the lockdown. Column (3) shows estimates when adding controls from the daily number of COVID-19 cases for the city of San Francisco and neighborhood by time fixed effects. Results in this column indicate a reduction of about 17% in the daily number of crimes after the lockdown. Finally, column (4) shows estimates when adding weather controls and their temporal lags. This is our most conservative specification. Here, we find that the SIP policy reduced total daily crime activity by 13.2% with respect to its historical trend. Results are well below in magnitude compared to most literature focusing on the effects of COVID-19 related restrictions on criminal activities.

### Impact of the shelter-in-place order on different crime categories

Figure 1 visualizes the change in total daily the number of crimes for both San Francisco and Oakland before and after introducing the shelter-in-place order on March 16. Although Oakland had a higher crime rate per capita than San Francisco before the policy intervention, reducing the number of crimes after the quarantine order is universal across both cities’ neighborhoods.

According to the Oakland Police Department Data, the city had 1.4 criminal incidents per 1000 inhabitants per day on average, with the western side being generally more dangerous than the eastern one. The two weeks following the forced quarantine implementation showed an average daily crime rate of just 0.7 with a reduction of about 50%. Similar conclusions are drawn for San Francisco, which declined by more than 40%.

**Table 3:** The impact of the shelter-in-place order on the number of criminal incidents

	Dep. variable: in (total crimes)							
	Assaults	Thefts	Drug	Sex	Domestic	Traffic	Weapon	Other
$$I^{post}$$	– 0.102$$***$$	– 0.122$$***$$	– 0.124$$***$$	– 0.085$$**$$	0.009	– 0.147$$***$$	– 0.151$$***$$	– 0.164$$***$$
	(0.033)	(0.024)	(0.026)	(0.036)	(0.010)	(0.028)	(0.025)	(0.037)
$$W \cdot Crimes$$	0.030$$***$$	0.031$$***$$	0.028$$***$$	0.023$$***$$	0.020$$***$$	0.027$$***$$	0.031$$***$$	0.030$$***$$
	(0.004)	(0.004)	(0.003)	(0.002)	(0.001)	(0.003)	(0.004)	(0.003)
Day of week FE	Yes	Yes	Yes	Yes	Yes	Yes	Yes	Yes
Time FE	Yes	Yes	Yes	Yes	Yes	Yes	Yes	Yes
Covid-19 cases	Yes	Yes	Yes	Yes	Yes	Yes	Yes	Yes
Neighborhood $\times$ year FE	Yes	Yes	Yes	Yes	Yes	Yes	Yes	Yes
Weather controls	Yes	Yes	Yes	Yes	Yes	Yes	Yes	Yes
Observations	802	148,662	27,051	8595	7823	40,598	29,589	8418

Inclusion of spatial and temporal dependence

This table presents results of the effects of social distancing measures on the natural logarithm of total crimes after one month from the introduction of the SIP in San Francisco. The model includes spatial and temporal dependence. Significant at $$***$$*p* < 0.01, $$**$$*p* < 0.05, $$*$$*p* < 0.1

In San Francisco, as shown in Table 3, thefts and assaults have dropped by 12% and 10% accordingly, compared to the historical trend in the same period. As few people were driving on the roads, the traffic accidents fell by about 15% in the same period.

Overall, the reduction in the number of crimes can be considered one of the positive side effects of the socially restrictive measures to stop the spread of the COVID-19 virus (Fig. 2). On the other hand, as people were required to stay home, domestic violence rates did not drop during the pandemic (Fig. 3).

## Conclusion

In this paper, we investigated the impact of the COVID-19 quarantine measures on the number of crimes in two major cities in the US. To examine the effects of the forced lockdown, we use the introduction of such a policy in the San Francisco Bay Area on March 16 as the source of time variation, coupled with the daily number of reported incidents for the cities of Oakland and San Francisco. Our analysis of these two major U.S. cities at the grid level could be viewed as a stepping stone in compiling a more comprehensive set of data to analyse the change in crime rates across city, county, and state borders considering the interaction between subnational autonomy and federal authority (Chiara 2021).

Our empirical strategy compares the number of incidents for each neighborhood of these two cities before and after the implementation of the policy under the assumption that trends in outcomes would have been similar in the absence of the restrictive measures. We provide evidence supporting the plausibility of this assumption by including a set of controls such as the day of the week, neighborhood by time fixed effects, and weather controls.

The results suggest an immediate drop of between 10 and 20 percentage points across both cities and crime types within a month after the introduction of the lockdown. However, we find that although crimes in San Francisco slowly increased as the restriction eased, even after the end of the SIP policy and the consequent return to normal life, they were slightly below their pre-covid levels. Concerning different types of crime, while theft, homicide, and traffic accidents have fallen sharply, domestic violence incidents show no reduction from our most conservative specification.

## Footnotes

1 The authors also show that the number of crimes then returned to pre-pandemic levels before the end of 2020.

2 Incidents are categorized according to the Uniformed Crime Report of the FBI. Categories range from arson to assault to sex offenses, and thefts.

3 Since early March, the voluntary lockdown was adopted by an increasing portion of people living in San Francisco (CNBC 2020).

4 Neighborhoods in San Francisco and Oakland significantly differ in average the number of crimes during the year (San Francisco Police Department 2020).
